# 2-Allyl-7-nitro-2*H*-indazole

**DOI:** 10.1107/S1600536813026743

**Published:** 2013-10-02

**Authors:** Assoman Kouakou, El Mostapha Rakib, Domenico Spinelli, Mohamed Saadi, Lahcen El Ammari

**Affiliations:** a Laboratoire de Chimie Organique et Analytique, Université Sultan Moulay Slimane, Faculté des Sciences et Techniques, Béni-Mellal, BP 523, Morocco; b Dipartimento di Chimica ’G. Ciamician’, Università degli Studi di Bologna, Via Selmi 2, I-40126 Bologna, Italy; c Laboratoire de Chimie du Solide Appliquée, Faculté des Sciences, Université Mohammed V-Agdal, Avenue Ibn Battouta, BP 1014, Rabat, Morocco

## Abstract

The asymmetric unit of the title compound, C_10_H_9_N_3_O_2_, contains two independent mol­ecules linked by a C—H⋯N hydrogen bond. Each mol­ecule has a similar conformation, being built up from fused five- and six-membered rings, each linked to an ally and nitro group, respectively. The indazole ring system makes dihedral angles of 2.7 (2) and 2.2 (2)°, respectively, with the plane through the nitro group. The allyl group is nearly perpendicular to the indazole system, as indicated by the N—N—C—C torsion angles of −75.3 (2) and −82.2 (2)°, this being the most important difference between the conformations of the two mol­ecules. In the crystal, mol­ecules are linked by C—H⋯O and π–π [inter-centroid distance = 3.6225 (8) Å] inter­actions to form a three-dimensional network.

## Related literature

For pharmacological effects of indazole derivatives, see: Baraldi *et al.* (2001 ▶); Li *et al.* (2003 ▶); Lee *et al.* (2001 ▶); Rodgers *et al.* (1996 ▶); Schmidt *et al.* (2008 ▶). For similar compounds, see: El Brahmi *et al.* (2012 ▶); Chicha *et al.* (2013 ▶).
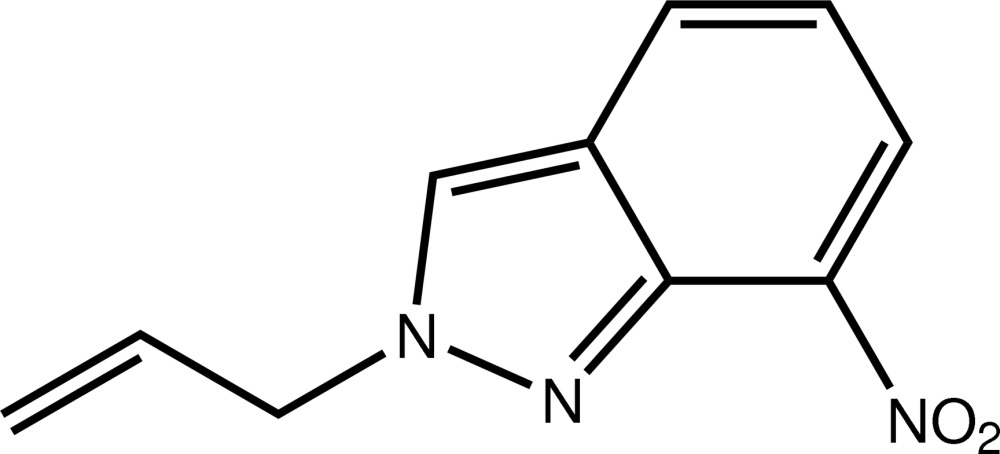


## Experimental

### 

#### Crystal data

C_10_H_9_N_3_O_2_
*M* *_r_* = 203.20
Triclinic, 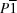

*a* = 8.1848 (3) Å
*b* = 8.3253 (4) Å
*c* = 16.3194 (6) Å
α = 84.168 (2)°
β = 85.653 (2)°
γ = 60.843 (2)°
*V* = 965.64 (7) Å^3^
*Z* = 4
Mo *K*α radiation
μ = 0.10 mm^−1^
*T* = 296 K
0.42 × 0.29 × 0.17 mm

#### Data collection

Bruker X8 APEX diffractometer
22310 measured reflections
4980 independent reflections
4107 reflections with *I* > 2σ(*I*)
*R*_int_ = 0.028

#### Refinement

*R*[*F* ^2^ > 2σ(*F* ^2^)] = 0.046
*wR*(*F* ^2^) = 0.142
*S* = 1.04
4980 reflections
271 parameters
H-atom parameters constrained
Δρ_max_ = 0.37 e Å^−3^
Δρ_min_ = −0.24 e Å^−3^

### 

Data collection: *APEX2* (Bruker, 2009 ▶); cell refinement: *SAINT* (Bruker, 2009 ▶); data reduction: *SAINT*; program(s) used to solve structure: *SHELXS97* (Sheldrick, 2008 ▶); program(s) used to refine structure: *SHELXL97* (Sheldrick, 2008 ▶); molecular graphics: *ORTEP-3 for Windows* (Farrugia, 2012 ▶); software used to prepare material for publication: *PLATON* (Spek, 2009 ▶) and *publCIF* (Westrip, 2010 ▶).

## Supplementary Material

Crystal structure: contains datablock(s) I. DOI: 10.1107/S1600536813026743/tk5258sup1.cif

Structure factors: contains datablock(s) I. DOI: 10.1107/S1600536813026743/tk5258Isup2.hkl

Supplementary material file. DOI: 10.1107/S1600536813026743/tk5258Isup3.cml

Additional supplementary materials:  crystallographic information; 3D view; checkCIF report

## Figures and Tables

**Table 1:** Hydrogen-bond geometry (Å, °)

*D*—H⋯*A*	*D*—H	H⋯*A*	*D*⋯*A*	*D*—H⋯*A*
C10—H10*A*⋯N2	0.93	2.60	2.907 (3)	100
C5—H5⋯O1^i^	0.93	2.49	3.4004 (19)	165
C8—H8*A*⋯O4^ii^	0.97	2.45	3.205 (2)	134
C15—H15⋯O4^i^	0.93	2.49	3.3986 (19)	167

Symmetry codes: (i) 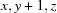; (ii) 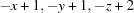.

**Fig. 1.**
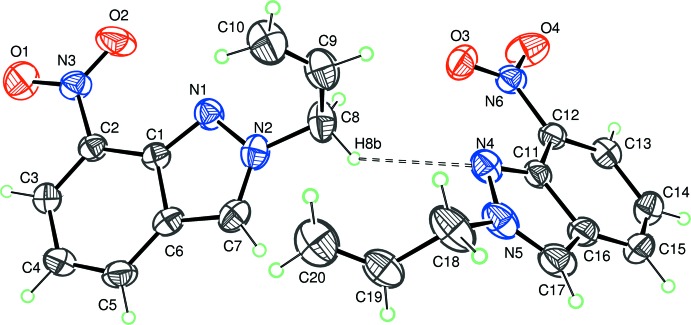
The two molecules comprising the asymmetric unit of the title compound with the atom-labelling scheme. Displacement ellipsoids are drawn at the 50% probability level. H atoms are represented as small circles.

**Fig. 2.**
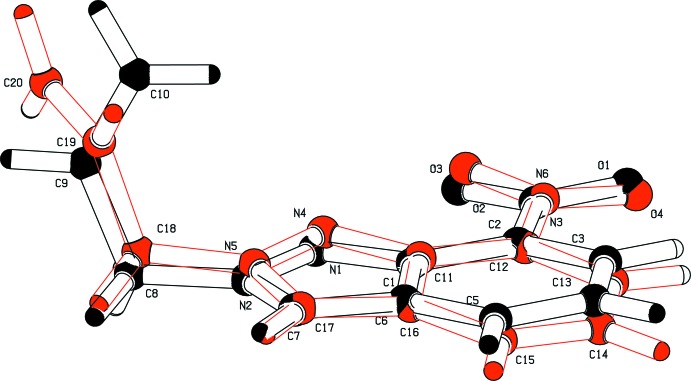
Overlay diagram of the two crystallographically independent molecules highlighting the different orientations of the allyl groups.

## supplementary crystallographic information

### 1. Comment

The indazole subunit in organic molecules is an important structure in many drug substances with a wide range of pharmacological effects: *e.g.*, anti-tumor, anti-microbial, anti-platelet, anti-HIV, and anti-inflammatory (Baraldi *et al.*, 2001; Li *et al.*, 2003; Lee *et al.*, 2001; Rodgers *et al.*, 1996; Schmidt *et al.*, 2008). The present work is a continuation of the investigation of the indazole derivatives published recently by our team (El Brahmi *et al.*, 2012; Chicha *et al.*, 2013).

The plot of the structure of the title compound, with two molecules in the asymmetric unit, shows them linked by a C8—H8B···N4 hydrogen bond, Fig. 1. In the molecules, the allyl groups are nearly perpendicular to indazole planes as indicated by the torsion angles of C8–C9–N1–N2 = -75.3 (2)° and C18–C19–N4–N5 = -82.2 (2)°. This is the most important difference between the two conformations of the molecules as shown in the overlay diagram of the two crystallographically independent molecules (Fig. 2). The dihedral angles of 2.7 (2) and 2.2 (2)°, respectively, between the fused ring systems and the nitro groups lead to a synperiplanar conformation for each molecule.

In the crystal, molecules are linked by C—H···O (Table 2) and π—π [inter-centroid distances between centrosymmetrically related (C1–C6) rings = 3.6225 (8) Å; symmetry operation = 1-*x*, 1-*y*, 1-*z*] interactions, forming a three-dimensional network.

### 2. Experimental

To a solution of 7-nitroindazole (6.13 mmol) in acetone (15 ml) was added potassium hydroxide (6.8 mmol). After 15 min. at 298 K, allyl bromide (12.26 mmol) was added drop wise. Upon disappearance of the starting material as indicated by TLC, the resulting mixture was evaporated. The crude material was dissolved with EtOAc (50 ml), washed with water and brine, dried over MgSO_4_ and the solvent was evaporated *in vacuo*. The resulting residue was purified by column chromatography (EtOAc/hexane 3/7). The title compound was recrystallized from ethanol at room temperature giving colorless crystals (m.p. 358 (1) K, yield: 65%).

### 3. Refinement

H atoms were located in a difference map and treated as riding with C—H = 0.93–0.97 Å, and with *U*_iso_(H) = 1.2*U*_eq_.

### Crystal data

**Table:**

C_10_H_9_N_3_O_2_	*Z* = 4
*M**_r_* = 203.20	*F*(000) = 424
Triclinic, *P*1	*D*_x_ = 1.398 Mg m^−^^3^
Hall symbol: -P 1	Mo *K*α radiation, λ = 0.71073 Å
*a* = 8.1848 (3) Å	Cell parameters from 4980 reflections
*b* = 8.3253 (4) Å	θ = 2.5–28.7°
*c* = 16.3194 (6) Å	µ = 0.10 mm^−^^1^
α = 84.168 (2)°	*T* = 296 K
β = 85.653 (2)°	Irregular shape, colourless
γ = 60.843 (2)°	0.42 × 0.29 × 0.17 mm
*V* = 965.64 (7) Å^3^	

### Data collection

**Table:**

Bruker X8 APEX diffractometer	4107 reflections with *I* > 2σ(*I*)
Radiation source: fine-focus sealed tube	*R*_int_ = 0.028
Graphite monochromator	θ_max_ = 28.7°, θ_min_ = 2.5°
φ and ω scans	*h* = −11→11
22310 measured reflections	*k* = −11→11
4980 independent reflections	*l* = −22→22

### Refinement

**Table:**

Refinement on *F*^2^	Primary atom site location: structure-invariant direct methods
Least-squares matrix: full	Secondary atom site location: difference Fourier map
*R*[*F*^2^ > 2σ(*F*^2^)] = 0.046	Hydrogen site location: inferred from neighbouring sites
*wR*(*F*^2^) = 0.142	H-atom parameters constrained
*S* = 1.04	*w* = 1/[σ^2^(*F*_o_^2^) + (0.076*P*)^2^ + 0.1973*P*] where *P* = (*F*_o_^2^ + 2*F*_c_^2^)/3
4980 reflections	(Δ/σ)_max_ = 0.001
271 parameters	Δρ_max_ = 0.37 e Å^−^^3^
0 restraints	Δρ_min_ = −0.24 e Å^−^^3^

### Special details

**Table:**

Geometry. All e.s.d.'s (except the e.s.d. in the dihedral angle between two l.s. planes) are estimated using the full covariance matrix. The cell e.s.d.'s are taken into account individually in the estimation of e.s.d.'s in distances, angles and torsion angles; correlations between e.s.d.'s in cell parameters are only used when they are defined by crystal symmetry. An approximate (isotropic) treatment of cell e.s.d.'s is used for estimating e.s.d.'s involving l.s. planes.
Refinement. Refinement of *F*^2^ against all reflections. The weighted *R*-factor *wR* and goodness of fit *S* are based on *F*^2^, conventional *R*-factors *R* are based on *F*, with *F* set to zero for negative *F*^2^. The threshold expression of *F*^2^ > 2σ(*F*^2^) is used only for calculating *R*-factors(gt) *etc*. and is not relevant to the choice of reflections for refinement. *R*-factors based on *F*^2^ are statistically about twice as large as those based on *F*, and *R*- factors based on all data will be even larger.

### Fractional atomic coordinates and isotropic or equivalent isotropic displacement parameters (Å^2^)

**Table:**

	*x*	*y*	*z*	*U*_iso_*/*U*_eq_	
C1	0.40636 (16)	0.52858 (16)	0.63233 (7)	0.0352 (2)	
C2	0.32039 (17)	0.46916 (16)	0.57894 (7)	0.0365 (3)	
C3	0.22059 (19)	0.58263 (18)	0.51389 (8)	0.0439 (3)	
H3	0.1656	0.5413	0.4795	0.053*	
C4	0.1994 (2)	0.76114 (19)	0.49786 (9)	0.0506 (3)	
H4	0.1297	0.8363	0.4534	0.061*	
C5	0.2794 (2)	0.82492 (18)	0.54647 (9)	0.0491 (3)	
H5	0.2659	0.9425	0.5354	0.059*	
C6	0.38287 (18)	0.71050 (17)	0.61358 (8)	0.0406 (3)	
C7	0.4834 (2)	0.7291 (2)	0.67340 (9)	0.0478 (3)	
H7	0.4978	0.8314	0.6792	0.057*	
C8	0.6739 (2)	0.5268 (3)	0.79150 (9)	0.0568 (4)	
H8A	0.6289	0.4718	0.8366	0.068*	
H8B	0.6606	0.6404	0.8092	0.068*	
C9	0.8763 (2)	0.3989 (3)	0.77569 (10)	0.0595 (4)	
H9	0.9568	0.3830	0.8168	0.071*	
C10	0.9523 (3)	0.3085 (3)	0.71208 (12)	0.0699 (5)	
H10A	0.8786	0.3191	0.6690	0.084*	
H10B	1.0812	0.2321	0.7089	0.084*	
N1	0.51163 (16)	0.44398 (16)	0.69883 (7)	0.0424 (3)	
N2	0.55488 (16)	0.57195 (17)	0.72082 (7)	0.0463 (3)	
N3	0.33824 (17)	0.28617 (16)	0.59100 (8)	0.0469 (3)	
O1	0.2663 (3)	0.24031 (19)	0.54180 (9)	0.0862 (5)	
O2	0.42204 (19)	0.18615 (16)	0.65005 (8)	0.0689 (3)	
C11	0.77659 (16)	0.77408 (16)	0.98570 (7)	0.0358 (3)	
C12	0.71532 (16)	0.68123 (16)	1.04771 (7)	0.0357 (3)	
C13	0.64456 (19)	0.75454 (19)	1.12190 (8)	0.0444 (3)	
H13	0.6060	0.6915	1.1621	0.053*	
C14	0.6294 (2)	0.9241 (2)	1.13810 (10)	0.0543 (4)	
H14	0.5804	0.9718	1.1888	0.065*	
C15	0.6852 (2)	1.0194 (2)	1.08103 (10)	0.0541 (4)	
H15	0.6741	1.1317	1.0922	0.065*	
C16	0.75989 (19)	0.94553 (17)	1.00491 (9)	0.0437 (3)	
C17	0.8334 (2)	1.0005 (2)	0.93490 (10)	0.0543 (4)	
H17	0.8441	1.1072	0.9265	0.065*	
C18	0.9745 (2)	0.8661 (3)	0.80039 (10)	0.0671 (5)	
H18A	1.0708	0.7405	0.7924	0.081*	
H18B	1.0348	0.9421	0.7980	0.081*	
C19	0.8404 (2)	0.9320 (2)	0.73243 (10)	0.0573 (4)	
H19	0.7356	1.0480	0.7344	0.069*	
C20	0.8612 (3)	0.8364 (3)	0.67013 (12)	0.0725 (5)	
H20A	0.9647	0.7199	0.6665	0.087*	
H20B	0.7726	0.8848	0.6294	0.087*	
N4	0.85309 (16)	0.72904 (16)	0.90988 (7)	0.0434 (3)	
N5	0.88521 (17)	0.87130 (18)	0.88237 (8)	0.0505 (3)	
N6	0.72571 (15)	0.50676 (15)	1.03441 (7)	0.0415 (2)	
O3	0.78980 (18)	0.43963 (15)	0.96842 (7)	0.0602 (3)	
O4	0.67488 (19)	0.43012 (16)	1.09090 (8)	0.0691 (4)	

### Atomic displacement parameters (Å^2^)

**Table:**

	*U*^11^	*U*^22^	*U*^33^	*U*^12^	*U*^13^	*U*^23^
C1	0.0349 (5)	0.0344 (5)	0.0368 (6)	−0.0177 (5)	0.0018 (4)	−0.0023 (4)
C2	0.0381 (6)	0.0340 (5)	0.0391 (6)	−0.0191 (5)	0.0006 (5)	−0.0028 (5)
C3	0.0472 (7)	0.0438 (7)	0.0408 (6)	−0.0213 (6)	−0.0050 (5)	−0.0044 (5)
C4	0.0571 (8)	0.0405 (7)	0.0450 (7)	−0.0172 (6)	−0.0076 (6)	0.0049 (5)
C5	0.0575 (8)	0.0323 (6)	0.0537 (8)	−0.0198 (6)	0.0006 (6)	0.0000 (5)
C6	0.0429 (6)	0.0349 (6)	0.0467 (7)	−0.0207 (5)	0.0035 (5)	−0.0073 (5)
C7	0.0499 (7)	0.0449 (7)	0.0560 (8)	−0.0275 (6)	0.0010 (6)	−0.0124 (6)
C8	0.0510 (8)	0.0786 (10)	0.0469 (8)	−0.0337 (8)	−0.0057 (6)	−0.0141 (7)
C9	0.0516 (8)	0.0773 (11)	0.0546 (9)	−0.0345 (8)	−0.0082 (7)	−0.0023 (8)
C10	0.0568 (9)	0.0864 (13)	0.0607 (10)	−0.0308 (9)	−0.0006 (8)	−0.0034 (9)
N1	0.0444 (6)	0.0458 (6)	0.0413 (6)	−0.0250 (5)	−0.0048 (4)	−0.0006 (5)
N2	0.0453 (6)	0.0548 (7)	0.0453 (6)	−0.0280 (5)	−0.0021 (5)	−0.0107 (5)
N3	0.0516 (6)	0.0404 (6)	0.0563 (7)	−0.0284 (5)	−0.0044 (5)	−0.0006 (5)
O1	0.1353 (13)	0.0694 (8)	0.0879 (9)	−0.0731 (9)	−0.0361 (9)	0.0031 (7)
O2	0.0785 (8)	0.0473 (6)	0.0881 (9)	−0.0373 (6)	−0.0305 (7)	0.0222 (6)
C11	0.0350 (5)	0.0359 (6)	0.0385 (6)	−0.0185 (5)	−0.0107 (4)	0.0035 (5)
C12	0.0354 (6)	0.0328 (5)	0.0394 (6)	−0.0171 (5)	−0.0079 (5)	0.0029 (4)
C13	0.0454 (7)	0.0467 (7)	0.0396 (6)	−0.0214 (6)	−0.0047 (5)	0.0012 (5)
C14	0.0592 (8)	0.0515 (8)	0.0484 (8)	−0.0214 (7)	−0.0056 (6)	−0.0131 (6)
C15	0.0601 (9)	0.0393 (7)	0.0652 (9)	−0.0229 (6)	−0.0165 (7)	−0.0084 (6)
C16	0.0457 (7)	0.0371 (6)	0.0532 (7)	−0.0236 (5)	−0.0165 (6)	0.0061 (5)
C17	0.0574 (8)	0.0509 (8)	0.0655 (9)	−0.0363 (7)	−0.0199 (7)	0.0172 (7)
C18	0.0548 (9)	0.0939 (13)	0.0546 (9)	−0.0423 (9)	−0.0056 (7)	0.0238 (9)
C19	0.0529 (8)	0.0657 (9)	0.0513 (8)	−0.0303 (7)	−0.0036 (6)	0.0144 (7)
C20	0.0687 (11)	0.0900 (13)	0.0579 (10)	−0.0402 (10)	0.0091 (8)	0.0004 (9)
N4	0.0441 (6)	0.0490 (6)	0.0400 (5)	−0.0257 (5)	−0.0059 (4)	0.0048 (5)
N5	0.0500 (6)	0.0615 (7)	0.0476 (6)	−0.0354 (6)	−0.0112 (5)	0.0162 (5)
N6	0.0421 (5)	0.0377 (5)	0.0482 (6)	−0.0223 (5)	−0.0070 (5)	0.0026 (4)
O3	0.0892 (8)	0.0508 (6)	0.0500 (6)	−0.0399 (6)	−0.0076 (5)	−0.0067 (5)
O4	0.0824 (8)	0.0530 (6)	0.0815 (8)	−0.0439 (6)	0.0186 (7)	0.0003 (6)

### Geometric parameters (Å, º)

**Table:**

C1—N1	1.3459 (17)	C11—N4	1.3459 (16)
C1—C2	1.4208 (17)	C11—C12	1.4196 (17)
C1—C6	1.4337 (17)	C11—C16	1.4313 (17)
C2—C3	1.3648 (18)	C12—C13	1.3686 (18)
C2—N3	1.4517 (16)	C12—N6	1.4509 (16)
C3—C4	1.409 (2)	C13—C14	1.407 (2)
C3—H3	0.9300	C13—H13	0.9300
C4—C5	1.358 (2)	C14—C15	1.358 (2)
C4—H4	0.9300	C14—H14	0.9300
C5—C6	1.402 (2)	C15—C16	1.403 (2)
C5—H5	0.9300	C15—H15	0.9300
C6—C7	1.389 (2)	C16—C17	1.391 (2)
C7—N2	1.330 (2)	C17—N5	1.326 (2)
C7—H7	0.9300	C17—H17	0.9300
C8—N2	1.4649 (19)	C18—N5	1.469 (2)
C8—C9	1.489 (2)	C18—C19	1.486 (2)
C8—H8A	0.9700	C18—H18A	0.9700
C8—H8B	0.9700	C18—H18B	0.9700
C9—C10	1.279 (2)	C19—C20	1.304 (3)
C9—H9	0.9300	C19—H19	0.9300
C10—H10A	0.9300	C20—H20A	0.9300
C10—H10B	0.9300	C20—H20B	0.9300
N1—N2	1.3615 (16)	N4—N5	1.3605 (16)
N3—O2	1.2137 (16)	N6—O3	1.2240 (15)
N3—O1	1.2207 (17)	N6—O4	1.2292 (15)
			
N1—C1—C2	131.9 (2)	N4—C11—C12	131.5 (2)
N1—C1—C6	111.6 (2)	N4—C11—C16	111.7 (2)
C2—C1—C6	116.5 (2)	C12—C11—C16	116.8 (2)
C3—C2—C1	120.6 (2)	C13—C12—C11	120.6 (2)
C3—C2—N3	118.2 (2)	C13—C12—N6	118.2 (2)
C1—C2—N3	121.3 (2)	C11—C12—N6	121.2 (2)
C2—C3—C4	121.2 (2)	C12—C13—C14	120.8 (2)
C2—C3—H3	119.4	C12—C13—H13	119.6
C4—C3—H3	119.4	C14—C13—H13	119.6
C5—C4—C3	120.8 (2)	C15—C14—C13	121.2 (2)
C5—C4—H4	119.6	C15—C14—H14	119.4
C3—C4—H4	119.6	C13—C14—H14	119.4
C4—C5—C6	118.9 (2)	C14—C15—C16	118.8 (2)
C4—C5—H5	120.6	C14—C15—H15	120.6
C6—C5—H5	120.6	C16—C15—H15	120.6
C7—C6—C5	134.0 (2)	C17—C16—C15	134.4 (2)
C7—C6—C1	104.0 (2)	C17—C16—C11	103.8 (2)
C5—C6—C1	121.9 (2)	C15—C16—C11	121.8 (2)
N2—C7—C6	106.6 (2)	N5—C17—C16	106.7 (2)
N2—C7—H7	126.7	N5—C17—H17	126.6
C6—C7—H7	126.7	C16—C17—H17	126.6
N2—C8—C9	114.9 (2)	N5—C18—C19	113.1 (2)
N2—C8—H8A	108.5	N5—C18—H18A	109.0
C9—C8—H8A	108.5	C19—C18—H18A	109.0
N2—C8—H8B	108.5	N5—C18—H18B	109.0
C9—C8—H8B	108.5	C19—C18—H18B	109.0
H8A—C8—H8B	107.5	H18A—C18—H18B	107.8
C10—C9—C8	127.5 (2)	C20—C19—C18	123.8 (2)
C10—C9—H9	116.3	C20—C19—H19	118.1
C8—C9—H9	116.3	C18—C19—H19	118.1
C9—C10—H10A	120.0	C19—C20—H20A	120.0
C9—C10—H10B	120.0	C19—C20—H20B	120.0
H10A—C10—H10B	120.0	H20A—C20—H20B	120.0
C1—N1—N2	103.1 (2)	C11—N4—N5	103.0 (2)
C7—N2—N1	114.7 (2)	C17—N5—N4	114.8 (2)
C7—N2—C8	126.3 (2)	C17—N5—C18	126.9 (2)
N1—N2—C8	119.0 (2)	N4—N5—C18	118.3 (2)
O2—N3—O1	122.8 (2)	O3—N6—O4	122.8 (2)
O2—N3—C2	118.7 (2)	O3—N6—C12	118.6 (2)
O1—N3—C2	118.5 (2)	O4—N6—C12	118.5 (2)

### Hydrogen-bond geometry (Å, º)

**Table:**

*D*—H···*A*	*D*—H	H···*A*	*D*···*A*	*D*—H···*A*
C10—H10*A*···N2	0.93	2.60	2.907 (3)	100
C5—H5···O1^i^	0.93	2.49	3.4004 (19)	165
C8—H8*A*···O4^ii^	0.97	2.45	3.205 (2)	134
C15—H15···O4^i^	0.93	2.49	3.3986 (19)	167

Symmetry codes: (i) *x*, *y*+1, *z*; (ii) −*x*+1, −*y*+1, −*z*+2.
